# Genome-wide analysis of long non-coding RNAs and mRNAs in lung adenocarcinoma with pulmonary thromboembolism

**DOI:** 10.3389/fgene.2025.1683576

**Published:** 2026-01-20

**Authors:** Maryamgvl Ahmat, Gvzalnur Alim, Zhu Zhu, Chao Chen, Guolei Cao, Qin Luo

**Affiliations:** 1 Respiratory and Neurology Department, The Affiliated Cancer Hospital of Xinjiang Medical University, Urumqi, Xinjiang, China; 2 Cardiothoracic Surgery Department, The Affiliated Cancer Hospital of Xinjiang Medical University, Urumqi, Xinjiang, China; 3 Cancer Research Institute, The Affiliated Cancer Hospital of Xinjiang Medical University, Urumqi, Xinjiang, China

**Keywords:** lung adenocarcinoma complicated by pulmonary thromboembolism, long non-coding RNA, mRNA, RNA sequencing, Kyoto Encyclopedia of Genes and Genome

## Abstract

**Introduction:**

Pulmonary thromboembolism (PTE) is a serious complication in patients with lung adenocarcinoma (LUAD), yet its molecular mechanisms remain poorly understood. This study aimed to investigate the expression profiles of long non-coding RNAs (lncRNAs) and mRNAs in LUAD patients complicated by PTE.

**Methods:**

Peripheral blood samples were collected from LUAD patients with PTE and from three control groups (LUAD-only, PTE-only, and healthy controls). RNA sequencing was performed to identify differentially expressed lncRNAs and mRNAs among groups.

**Results:**

RNA sequencing revealed significant dysregulation of transcripts. Compared with LUAD-only patients, 725 lncRNAs and 2,052 mRNAs were differentially expressed in the LUAD + PTE group. Compared with PTE-only patients, 932 lncRNAs and 2,206 mRNAs were differentially expressed, while comparison with healthy controls identified 1,190 lncRNAs and 3,001 mRNAs. Key dysregulated transcripts included MERGE.31027.6, ENST00000318988, MERGE.30976.2, and ENST00000397519. Enrichment analyses highlighted immune response–related pathways, cytokine signaling, and the NF-κB signaling pathway.

**Conclusion:**

These findings suggest that aberrant lncRNA and mRNA expression may contribute to the pathogenesis of LUAD complicated by PTE and may serve as potential biomarkers and therapeutic targets for prognosis and treatment.

## Introduction

Lung cancer is the most common cancer, accounting for the highest incidence and mortality rates globally. An estimated 2.4 million new cases and more than 1.8 million deaths occurred in 2020, with non-small-cell lung cancer accounting for more than 85% of cases and deaths (International Agency for Research on Cancer, 2024).

Pulmonary thromboembolism (PTE) is a common complication associated with lung cancer and has emerged as a leading cause of mortality in patients afflicted with the disease. Lung cancer patients with pulmonary embolism (PE) or unsuspected pulmonary embolism (UPE) exhibit a shorter mean survival than those without these conditions, with higher mortality in suspected PE cases than in confirmed UPE cases (Li et al., 2018). Some studies have shown that incidentally detected asymptomatic PE is an independent risk factor associated with 1-year mortality in lung cancer patients (Luo et al., 2023). Because the clinical symptoms of lung cancer complicated by PTE are atypical and heterogeneous, misdiagnosis or missed diagnosis is often common, which exacerbates the condition of patients with lung cancer and poses a significant clinical challenge. An increased risk of blood clot formation is associated with the presence of the tumor itself, surgical procedures, chemotherapy, tyrosine kinase inhibitors, and immunomodulatory drugs (Fujieda et al., 2021). In addition, there are concerns regarding the choice of the optimal anticoagulation strategy to avoid bleeding. Therefore, it would be beneficial to identify more risk factors that might help us foresee PTE formation in lung cancer patients.

Long non-coding are RNAs over 200 nt in length but lack obvious open reading frame and are unable to encode proteins. lncRNA is closely related to the regulation of biological processes in cells and the pathophysiology of human diseases (Heydari et al., 2024). Compared with the protein-coding genes (Zhao et al., 2023) and shorter non-coding RNA [such as miRNA (Seelan et al., 2022)], the repertoire of lncRNA is larger with more diverse and complex expression patterns (Zhang et al., 2021). To date, more than 50, 000 lncRNA genes have been cloned and identified in the human genome, but only a limited number of these have been functionally characterized in the context of cancer. Although functional studies are not widely conducted, lncRNAs have been proven to demonstrate great potential as targets in treatment and as biomarkers in the diagnosis of malignant tumors and other human diseases (Masoud and Mohamadynejad, 2024). However, the potential role of lncRNAs in lung cancer patients with PTE has seldom been investigated despite its possible clinical relevance. Alternatively, the signaling pathways involved in PTE development in lung adenocarcinoma patients remain poorly defined. Considering the intricate array of risk factors associated with PTE, it is plausible to suggest that multiple signaling pathways are involved in its development. Clarifying the unique transcript profile may offer valuable insights into this critical issue.

The purpose of this study was to identify distinct lncRNA and mRNA expression patterns in the peripheral blood of patients with lung adenocarcinoma (LUAD) complicated by PTE. Herein, we compared the differentially expressed lncRNAs and mRNAs in the peripheral blood of patients with LUAD complicated by PTE and those with LUAD, PTE without cancer, or healthy subjects via Illumina high-throughput sequencing. The differentially expressed genes (DEGs) were subjected to Gene Ontology (GO) and Kyoto Encyclopedia of Genes and Genomes (KEGG) pathway enrichment analyses to identify lncRNAs and mRNAs that may be associated with the development of PTE in patients with LUAD, thereby providing evidence for potential novel biomarkers and therapeutic targets for the disease.

## Materials and methods

### Study subjects

This study was approved by the Ethics Committee of the Cancer Hospital Affiliated to Xinjiang Medical University. All participants provided written informed consent prior to enrollment. A total of 40 participants were recruited among patients admitted to our hospital between January 2019 and December 2019. All participants were divided into case group (n = 10): patients with LUAD complicated by PTE; control-1 (n = 10): patients with LUAD without PTE; control-2 (n = 10): patients with PTE without malignancy; and control-3 (n = 10): healthy controls without cancer, thrombosis, or other underlying diseases.

### Inclusion and exclusion criteria

Case group: LUAD diagnosed by histopathological biopsy; PTE confirmed by pulmonary artery computed tomography angiography (CTA); no history of cardiovascular disease, cerebrovascular disease, endocrine disorders, or other malignant tumors.

Control-1: Biopsy-proven LUAD (consistent with the case group) but without PTE (negative pulmonary artery CTA); no other exclusionary comorbidities.

Control-2: PTE confirmed by pulmonary artery CTA; no history of malignancy (confirmed by imaging and laboratory tests); no other exclusionary comorbidities.

Control-3: Healthy individuals undergoing routine physical examination; no evidence of thrombus, cardiovascular/cerebrovascular disease, endocrine disorders, or malignancy (based on medical history and physical examination).

### Blood sample, library preparation, and RNA sequencing

Peripheral blood samples of the 40 participants were collected in BD RNA blood tubes (Cat. #762165) prior to treatment: 10 with LUAD + PTE, 10 with LUAD-only, 10 with PTE-only, and 10 healthy controls (n = 10 biological replicates per group; no technical replicates). Total RNA was extracted using TRIzol® reagent (Invitrogen), quantified with the Qubit RNA BR Assay kit (Thermo Fisher Scientific), and ribosomal RNA (rRNA) depleted using the Ribo-Zero™ magnetic kit (Epicentre). RNA integrity was assessed using an Agilent RNA 6000 pico chip (Agilent Technologies), and only samples with RNA integrity number (RIN) ≥ 8 were used for library construction. Libraries were synthesized with SuperScript II reverse transcriptase (Invitrogen) following the manufacturer’s protocol, and sequencing was performed on an Illumina HiSeq 2500 platform with 150 bp paired-end reads. PCR duplicates were removed using Picard MarkDuplicates (v2.26.11) to retain only unique reads for downstream analysis.

### RNA sequencing data analysis

Fastp software was used for quality control (QC), adapter trimming, and low-quality read filtering of FASTQ data. Transcripts were assembled via StringTie, and lncRNAs were identified by combining coding potential calculator (CPC, score <0), coding–non-coding index (CNCI, score <0), and HMMER (E-value <0.001) to exclude protein-coding transcripts. A transcript was included in differential expression analysis if (1) its average expression was ≥0.5 in at least one group with more than two-thirds of samples expressing it or (2) its average expression was ≥1 in at least one group with more than two-thirds of samples expressing it.

Differential expression of lncRNAs and mRNAs between the LUAD + PTE group and each control group (LUAD-only, PTE-only, and healthy controls) was analyzed using limma and DESeq packages. Significance was defined as |log_2_ fold change (FC)| ≥ 1 and Benjamini–Hochberg false discovery rate (FDR) < 0.05 (FDR correction applied to control for multiple testing). Transcripts per kilobase million (TPM), FDR-adjusted p-values, FC, and expression differences across the four groups were summarized. Systematic clustering was used to generate heatmaps of differentially expressed known mRNAs, known lncRNAs, novel mRNAs, and novel lncRNAs. Target Finder software predicted lncRNA–mRNA target pairs, and GO and KEGG pathway enrichment analyses were performed to explore biological functions associated with LUAD + PTE. To compare the characteristics of lncRNAs and mRNAs, transcript length and exon number per transcript were quantified using the following steps: transcript length calculation: the genomic coordinates of assembled transcripts (from StringTie) were used to compute the length. Exon number counting: exon annotations from the GTF file generated using StringTie were parsed to count exons per transcript. Statistical comparison: distribution differences between lncRNAs and mRNAs were visualized using violin plots (length) and bar plots (exon number), and significance was assessed via the Mann–Whitney U test (two-tailed, p < 0.05).

### Statistical analysis

Correlation analysis between lncRNA and mRNA expression was performed using StringTie and prepDE.py to determine Pearson correlation coefficients (|r| ≤ 1). Clustering analysis of differentially expressed transcripts was visualized via heatmaps generated using the R package pheatmap.

## Results

### Identification of lncRNAs and mRNAs in the peripheral blood

**FIGURE 1:**
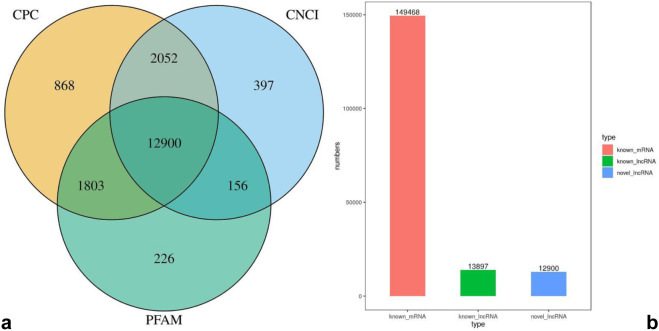
**(a)** Venn diagram illustrating the identification of novel lncRNAs using CPC, CNCI, and PFAM scores after the putative protein-coding transcripts were removed. **(b)** Number of identified mRNAs, known lncRNAs, and novel lncRNAs.

According to the lncRNA characteristics, transcripts shorter than 200 bp were excluded from lncRNA identification. Transcripts with a single exon or multiple exons were further filtered out if their fragments per kilobase of the exon model per million mapped reads (FPKM) were ≥2 or ≤0.5, respectively. To eliminate potential coding transcripts, three tools were combined: CPC score <0, CNCI score <0, and E-value <0.001. The intersection of 12,900 transcripts from these three tools was defined as novel lncRNAs (Figures 1a,b). Additionally, 13,897 known lncRNAs were identified via sequence alignment in the Ensembl database (release 112). For mRNA identification, transcripts were retained if they met the following criteria: (1) length ≥200 bp, (2) FPKM ≥1 in at least one sample group, and (3) coding potential confirmed by CPC score ≥0, CNCI score ≥0, and Pfam E-value ≥0.001 (opposite thresholds to lncRNA filtering). A total of 149,468 mRNAs were identified through this process (Figure 1b).

### Comparison of lncRNA and mRNA characteristics

**FIGURE 2:**
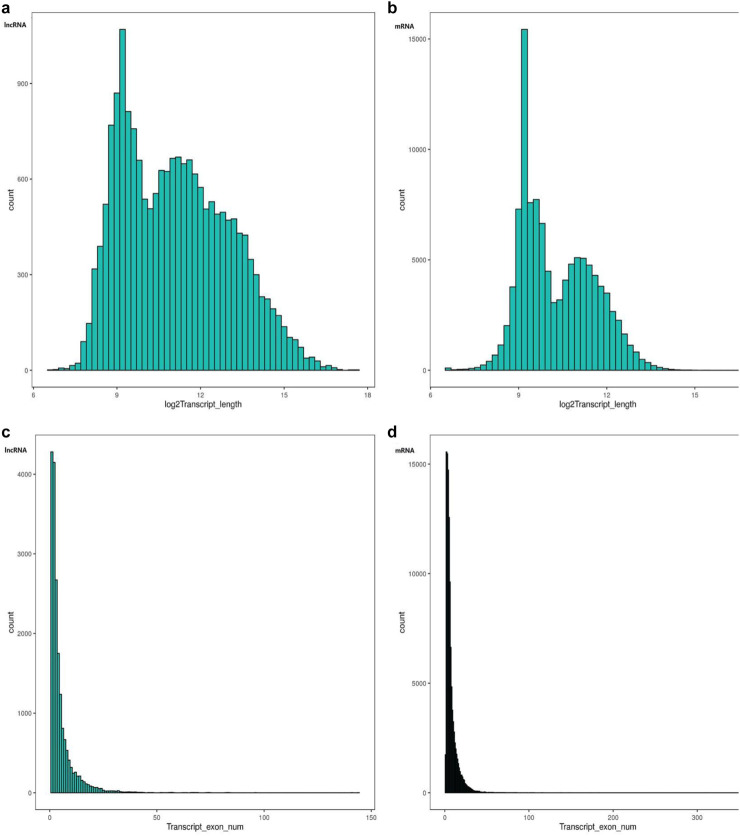
Structural characteristics of lncRNAs and mRNAs. **(a)** lncRNA transcript length distribution. **(b)** mRNA transcript length distribution. **(c)** lncRNA exon number distribution. **(d)** mRNA exon number distribution. Note: For **(a)** and **(b)**, the x-axis represents the transcript length (log_2_-transformed), and the y-axis represents the number of transcripts at corresponding lengths. For **(c)** and **(d)**, the x-axis represents the number of exons per transcript, and the y-axis represents the number of transcripts with corresponding exon counts. Statistical significance was determined using the Mann–Whitney U test (p < 0.05).

The characteristics of lncRNAs and mRNAs were compared in terms of transcript length and exon number per gene, with analyses performed using genomic coordinates and exon annotations from StringTie-assembled transcripts (Methods). lncRNAs exhibited a wider length distribution than mRNAs, but they had a significantly lower average length (Figures 2a,b). Notably, the maximum length of lncRNAs exceeded that of mRNAs. With regard to exon number, lncRNAs contained fewer exons per gene than mRNAs (Figures 2c,d).

### Identification of differentially expressed lncRNAs and mRNAs

**FIGURE 3:**
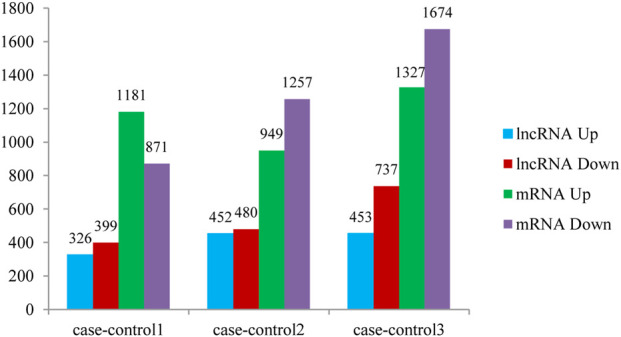
Histogram for the difference comparison of lncRNAs and mRNAs. Note: The x-axis represents pairwise comparisons between groups, and the y-axis represents the number of differentially expressed transcripts.

**FIGURE 4:**
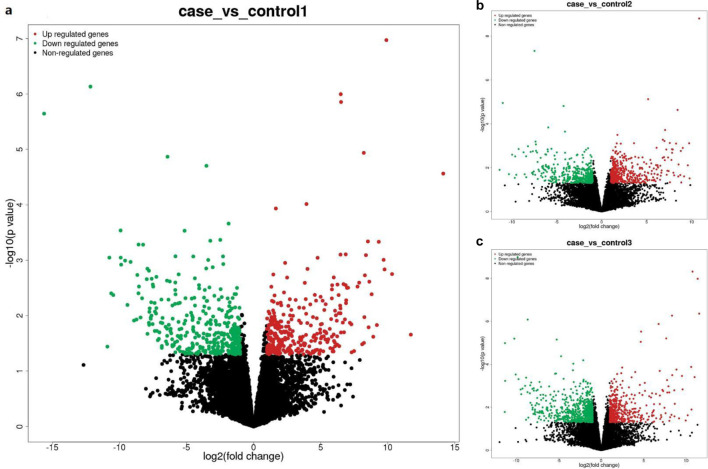
Volcano plot of differentially expressed lncRNAs. **(a)** Case vs. control-1, **(b)** case vs. control-2, and **(c)** case vs. control-3. Note: The x-axis represents log_2_ (fold change) of the expression levels, and the y-axis represents −log_10_ (adjusted p-value) from differential expression analysis. Red dots indicate upregulated lncRNAs, green dots indicate downregulated lncRNAs, and black dots indicate non-significantly differentially expressed lncRNAs. The same color scheme applies to the subsequent volcano plots.

**FIGURE 5:**
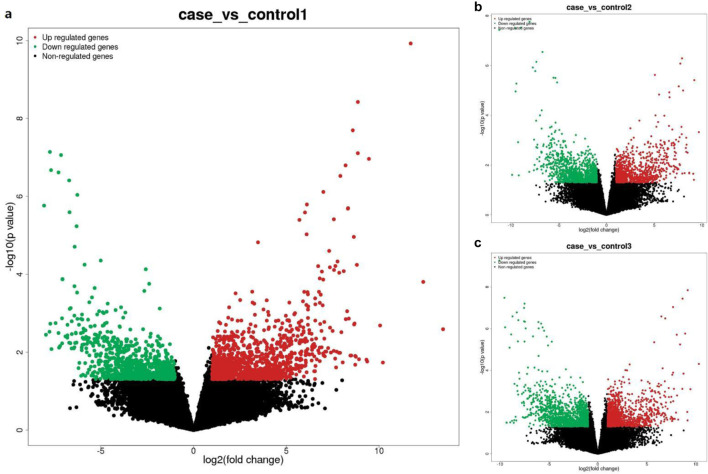
Volcano plots of differentially expressed mRNAs. **(a)** Case vs. control-1, **(b)** case vs. control-2, and **(c)** case vs. control-3.

Pairwise comparisons between the case group (LUAD with PTE) and each of the three control groups were performed to identify differentially expressed lncRNAs and mRNAs. The number of differentially expressed transcripts varied depending on the control group used for comparison (Figure 3). Case vs. control-1 (LUAD-only): this comparison yielded the fewest differentially expressed transcripts: 725 lncRNAs (326 upregulated and 399 downregulated) and 2,052 mRNAs (1,181 upregulated and 871 downregulated) (Figures 3, 4a, 5a). Case vs. control-3 (healthy controls): this comparison identified the most differentially expressed transcripts: 1,190 lncRNAs (453 upregulated and 737 downregulated) and 3,001 mRNAs (1,327 upregulated and 1,674 downregulated) (Figures 3, 4c, 5c). Case vs. control-2 (PTE-only): intermediate numbers of differentially expressed transcripts were observed in this case: 932 lncRNAs (452 upregulated and 480 downregulated) and 2,206 mRNAs (949 upregulated and 1,257 downregulated) (Figures 3, 4b, 5b).

These results indicate distinct transcriptional profiles in the case group when compared to different control groups. The top 10 upregulated and downregulated lncRNAs and mRNAs (sorted by fold change) from the case vs. healthy control comparison are presented in Tables 1, 2 (lncRNAs) and Tables 3, 4 (mRNAs), respectively.

**TABLE 1:** Top 10 upregulated lncRNAs.

ID	Log_2_ FC	p-value	pos_info	Gene name
MERGE.31027.6	11.535	<0.001	9:-:27534357–27573448	*CDKN2A-AS1*
MERGE.31027.13	11.357	<0.001	9:-:27546555–27573448	*CDKN2A-AS1*
MERGE.9105.9	10.988	<0.001	14:-:49545450–49862849	*FOXA1-AS*
MERGE.23375.6	10.75	<0.001	3:-:187722432–187745472	*LRP1B-IT1*
MERGE.26826.3	10.591	<0.001	6:-:31268749–31356905	*TERT-AS1*
MERGE.27409.2	9.504	<0.001	6:-:88279494–88442919	*HLA-A-AS*
MERGE.7659.2	8.856	<0.001	12:-:106237877–106247988	*KRAS-AS*
MERGE.17374.61	8.336	<0.001	19:-:54274466–54278544	*CEACAM19-AS1*
MERGE.19964.2	7.747	<0.001	20:-:20389552–20640806	*ERBB4-AS1*
MERGE.11060.20	7.648	<0.001	15:-:79898904–79923702	*IGF1R-AS*

**TABLE 2:** Top 10 downregulated lncRNAs.

ID	Log_2_ FC	p-value	pos_info	Gene name
MERGE.30976.2	9.842	<0.01	9:-:15464066–15510289	*PTENP1-AS*
MERGE.30701.1	9.735	<0.01	8:-:129819194–129939771	*MYC-AS1*
MERGE.9714.7	9.488	<0.01	14:-:92939862–93115279	*FOXP3-AS1*
MERGE.32224.4	9.414	<0.01	X:-:1367687–1392088	*AR-AS1*
MERGE.11753.14	9.265	<0.01	16:-:12744379–12803844	*BRCA1-AS*
MERGE.24085.17	8.957	<0.01	4:-:77728877–77819615	*FGFR1-AS*
MERGE.11724.23	8.853	<0.01	16:-:11548000–11586919	*CDH1-AS*
MERGE.24085.13	8.842	<0.01	4:-:77720238–77819615	*FGFR1-AS*
MERGE.21579.14	8.678	<0.01	22:+:40046955–40077033	*RB1-AS1*
MERGE.15786.11	8.661	<0.01	19:-:6417739–6424794	*CCNE1-AS*

**TABLE 3:** Top 10 upregulated mRNAs.

ID	Log_2_ FC	p-value	pos_info	Gene name
ENST00000318988	10.32	<0.001	17:-:7243839–7251961	*TP53*
ENST00000430436	9.455	<0.001	20:-:20389552–20639900	*ERBB2*
ENST00000341060	9.179	<0.001	6:-:24649979–24666591	*KRAS*
ENST00000377661	8.922	<0.001	5:+:154190774–154419031	*ALK*
ENST00000398812	8.748	<0.001	7:-:50590063–50732353	*BRAF*
ENST00000571980	8.65	<0.001	17:-:4272297–4366628	*LINC01503*
ENST00000251582	8.629	<0.001	5:-:179110851–179345430	*MET*
ENST00000267396	8.4	<0.001	14:+:22883165–22887686	*ALK*
ENST00000380262	8.059	<0.001	17:+:7932155–7949576	*TP53*
ENST00000367446	7.771	<0.001	1:+:193060034–193085985	*BRCA2*

**TABLE 4:** Top 10 downregulated mRNAs.

ID	Log_2_ FC	p-value	pos_info	Gene name
ENST00000397519	9.552	<0.001	9:-:15470646–15510289	*PTEN*
ENST00000409080	9.478	<0.001	2:+:172427789–172506282	*ERBB3*
ENST00000357674	9.02	<0.001	3:-:197042561–197298576	*PIK3R1*
ENST00000442218	8.859	<0.001	12:+:47773208–47782753	*MIR125B2*
ENST00000308478	8.721	<0.001	7:+:111091006–111124932	*NRAS*
ENST00000330714	8.519	<0.001	21:+:41361943–41408943	*PTGS2*
ENST00000418611	8.286	<0.001	3:+:45689241–45743804	*LINC00473*
ENST00000500704	8.243	<0.001	6:-:99432379–99515425	*MIR4660*
ENST00000646561	8.212	<0.001	20:-:472498–543835	*SNORD116-2*
ENST00000617047	8.17	<0.001	1:+:110339466–110346677	*Y_RNA*

Abbreviations: ID, assembled transcript ID; |log_2_ FC|, absolute value of log_2_ of the difference multiple; gene name, neighboring protein-coding gene; -AS, antisense lncRNA; -IT, intronic lncRNA; pos_info (position information, location information of the assembled transcripts on reference genomes.

### Enrichment analyses of target genes of lncRNAs

**FIGURE 6:**
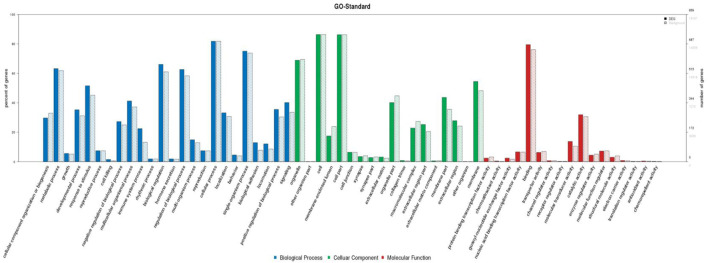
Secondary frequency diagram of GO. Note: The x-axis represents GO terms (biological process, cellular component, and molecular function), and the y-axis is split into two parts: the left side shows the percentage of genes annotated to each term, and the right side shows the absolute count of genes in each term.

To analyze the roles of differentially expressed lncRNAs involved in PTE formation among LUAD patients, we first predicted their target genes using Target Finder. Then we utilized GO analysis to analyze the functional roles of the target genes. The GO analysis identified several enriched genes (Figure 6). Among them, biological processes accounted for 6,018 terms, cellular components accounted for 666 terms, and molecular functions accounted for 1,243 terms. The majority of the 7,927 enriched terms were related to cellular processes and immune system response. The distribution of differential genes and background genes on each secondary entry in GO functional analysis is shown in Figure 6.

**FIGURE 7:**
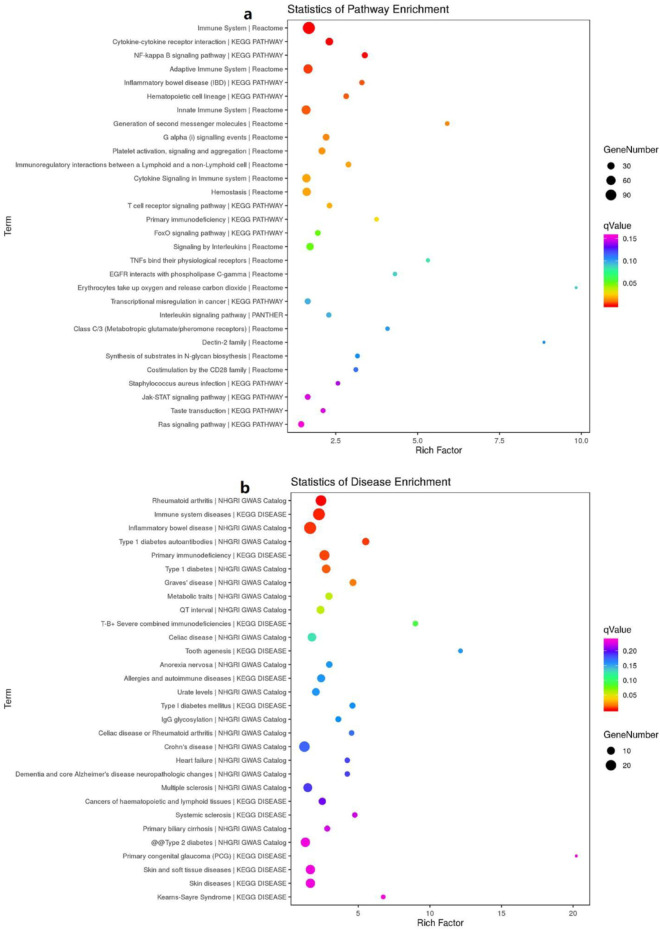
KEGG enrichment bubble map of differentially expressed genes. **(a)** KEGG pathway analysis of differentially expressed genes. **(b)** Analysis of KEGG disease enrichment of differentially expressed genes. Note: The x-axis represents the rich factor, and the y-axis represents the KEGG pathway/disease names. The size of the dots corresponds to the number of DEGs enriched in each pathway/disease, and the color gradient reflects the Q-value (FDR-adjusted p-value), with darker colors indicating lower Q-values (higher significance).

To investigate pathway enrichment of DEGs between the case group (LUAD with PTE) and control-3 (healthy controls), we used the KEGG Orthology-Based Annotation System (KOBAS 3.0) with default parameters. A KEGG enrichment scatter plot was constructed to visualize the pathways associated with DEGs, in which enrichment was quantified with the rich factor (ratio of DEGs enriched in a pathway to total genes in the pathway; higher values indicate greater enrichment), Q-value (FDR-adjusted p-value; values closer to 0 indicate higher significance), and the number of enriched genes (count of DEGs mapped to each pathway). KEGG analysis revealed 16 pathways that were significantly enriched in the case group (corrected p-value <0.05), including immune-related pathways (*immune system*, *adaptive immune system*, and *T-cell receptor signaling pathway*), inflammatory pathways (*cytokine–cytokine receptor interaction* and *NF-κB signaling pathway*), and hemostasis-related pathways (*platelet activation*, *signaling and aggregation*, and *hemostasis*). Additionally, several disease-related pathways were perturbed, such as *rheumatoid arthritis*, *inflammatory bowel disease*, and *type 1 diabetes* (Figure 7).

## Discussion

According to tumor statistics released by the National Cancer Society (American Cancer Society, ACS) in 2018, the incidence of lung cancer ranks second among both men and women, reaching 14% and 13%, respectively. In addition, lung cancer remains the leading of cancer-related deaths among men and women globally, accounting for 26% and 25%, respectively (American Cancer Society, 2025). PTE is a common complication of lung cancer, and has emerged as a leading cause of mortality in patients with lung cancer. The occurrence of PTE has significantly increased the mortality of patients with lung cancer (Howlett et al., 2020; Sørensen et al., 2023). At present, the pathogenesis of lung cancer complicated by PTE remains incompletely understood. It is generally believed that PTE is involved in the synthesis of various procoagulant substances and the secretion of vascular growth factors by lung cancer cells, which leads to hypercoagulation and activation of the coagulation system in patients with lung cancer (Stoencheva et al., 2023); however, the specific molecular mechanism remains to be elucidated. Therefore, the specific pathogenesis of lung cancer complicated by PTE needs to be further explored.

lncRNA refers to a class of functional non-coding RNA molecules with more than 200 bp nucleotides and no protein-coding function. Recent studies have shown that lncRNAs regulate gene expression at the epigenetic, transcriptional, and post-transcriptional levels. It is widely involved in many important regulatory processes, such as X chromosome silencing, genomic imprinting, chromatin modification, transcriptional activation, transcriptional interference, and intra-nuclear transport, among others. It is closely related to the occurrence, development, prevention, and treatment of human diseases (Taniue and Akimitsu, 2021; Much et al., 2024; Ferrer and Dimitrova, 2024; Herman et al., 2022). The abnormal expression of lncRNA is reported in the occurrence of a variety of diseases; for example, PCA3, a specifically expressed lncRNA of the prostate, has been used in the clinical diagnosis of prostate cancer because of its abnormal elevation in the urine of patients with prostate cancer (Wei et al., 2024). In addition, the elevated expression of lncRNAs GAS5, linc0597, and lncRNA-DC in the plasma of SLE patients has shown promise as a new diagnostic marker of SLE (El-Ashmawy et al., 2020). Therefore, differentially expressed lncRNAs in diseases have emerged as diagnostic markers of diseases and potential targets for novel therapy. In this study, the Illumina high-throughput sequencing technique was used to compare the differential expression of lncRNAs and mRNAs in LUAD complicated by PTE, pulmonary carcinoma, PTE, and normal population to search for the possible lncRNAs and mRNAs associated with LUAD complicated by PTE.

In this study, comparing LUAD + PTE vs. LUAD-only, a total of 725 differentially expressed lncRNAs and 2,052 differentially expressed mRNAs were identified. When comparing LUAD + PTE vs. PTE-only, a total of 932 differentially expressed lncRNAs and 2,206 differentially expressed mRNAs were identified. When comparing LUAD + PTE vs. healthy controls, a total of 1,190 differentially expressed lncRNAs and 3,001 differentially expressed mRNAs were identified. Among these, the upregulated lncRNA and mRNA with the largest difference multiple were MERGE.31027.6 and ENST00000318988, respectively. The downregulated lncRNA and mRNA with the largest difference multiple were MERGE.30976.2 and ENST00000397519, respectively. ENST00000318988 is also referred to as DULLARD, HSA011916, and NET56. Currently, it is believed to be involved in connective tissue diseases and nuclear envelope phosphatase 1. GO functional analysis showed that it was associated with cell mitotic cycle, protein binding, and other cellular activities. Some researchers have shown that DULLARD is a new negative regulator of TGF-β signaling in endochondral ossification (Rodrigues-Junior and Moustakas, 2024), while the increased expression of TGF-β can be detected in lung cancer (Konno et al., 2024). The reason for the significant difference in DULLARD (ENST00000318988) expression between LUAD complicated by PTE and the normal control group is attributed to the activities of the TGF-β signaling pathway. Activation of the TGF-β signaling pathway in patients with lung carcinoma, on the one hand, can promote the growth and metastasis of tumor cells. As tumor cells proliferate rapidly, the expansion of the vascular network cannot be adequately established to meet the demands of the increasing tumor volume; thus, tumor tissues develop immature and structurally abnormal vascular networks, leading to reduced oxygen availability, nutrient deprivation, and the accumulation of acidic substances in the microenvironment. On the other hand, the PI3k/Akt/HIF-1a signaling pathway is activated, and HIF-1a could regulate the expression of downstream VEGF, thereby promoting tumor angiogenesis. Meanwhile, TGF-β signaling is also directly related to the formation of vascular endothelial cells and thrombospondin S1 (THB-S1), which induces platelet aggregation and inhibits angiogenesis. Consequently, we hypothesize that that the activation of the TGF-β signaling pathway increased the responsiveness of DULLARD and was involved in the occurrence of PTE in patients with LUAD as the difference in DULLARD expression was not seen in the LUAD-only group and the PTE-only group compared with the normal controls. ENST00000397519 is also referred to as DFS70, LEDGF, PAIP, PSIP2, p52, and p75. The GO functional analysis showed that it was related to PC4 and SFRS1 interacting protein 1 (PSIP1). A previous study has shown that PSIP1 can help detect lung cancer with higher accuracy, particularly in early stages (Aftabi et al., 2024). In addition, PSIP1 could elicit humoral immune responses in lung cancer, and its autoantibodies have been reported to be correlated with tumorigenesis of lung cancer (Akele et al., 2025). We further compared the differentially expressed mRNAs between the LUAD-only and PTE-only groups and the normal control group and found a significant difference in ENST00000397519 expression between the LUAD-only and normal control groups (*p* < 0.001, |log_2_ FC| = 7.864). There were also differences between the PE-only group and the normal control group, but these were not statistically significant. According to our results and those from the previous research, we theorize that ENST00000397519 may serve as a potential marker for lung carcinoma screening. lncRNA-MERGE.31027.6 and lncRNA-MERGE.30976.2 were also significantly differentially expressed between the LUAD + PTE and normal control groups; however, it was not found in the relevant database. Nonetheless, based on the information obtained, we intend to conduct further research to verify its function and role in the occurrence of LUAD complicated by PTE.

GO and KEGG enrichment analyses revealed some pathways closely associated with LUAD complicated by PTE, such as immune system, cytokine–cytokine receptor interaction, NF-κB signaling pathway, adaptive immune system, inflammatory bowel disease (IBD), hematopoietic cell lineage, innate immune system, generation of second messenger molecules alpha (i) signaling events, and platelet activation. It is evident that most of the signaling pathways are involved in the dysregulation of the immune response. Several studies have provided insights into the critical and multiple roles of the immune system in the initiation and progression of diseases, while cancer and thrombosis are not an exception. A recent study provided evidence for the implication of the innate immune system in venous thrombosis (VTE) (Demoen et al., 2024). In addition, existence of a probable association between VTE and several other markers of inflammation such as C-reactive protein (CRP), IL-6, IL-8, and tumor necrosis factor alpha was demonstrated (Siddiqui et al., 2024; Setiawan et al., 2022; Oblitas et al., 2025; Imiela et al., 2024; Branchford and Carpenter, 2018). Our result well-corroborated the findings of those studies at the transcriptome level. In clinical processes, preventing the occurrence of thrombus is a critical consideration, particularly in the diagnosis and treatment of LUAD. Therefore, medical and nursing staff should use risk-assessment models, such as the Caprini scoring form and Padua scoring form, to evaluate the risk of VTE in patients to achieve timely diagnosis and treatment.

In conclusion, this study identified a profile of distinct lncRNAs and mRNAs in the peripheral blood of patients with LUAD complicated by PTE. Among these, MERGE.31027.6, MERGE.30976.2, ENST00000318988, and ENST00000397519 may be involved in the occurrence and development of LUAD complicated by PTE. In future work, we intend to further explore the roles of differentially expressed RNAs in the initiation and development of LUAD complicated by PTE, aimed at identifying novel targets for diagnosis and treatment. Meanwhile, it is suggested that medical and nursing staff should pay attention to immune system, cytokine–cytokine receptor interaction, and other related diseases as dysregulation in these pathways leads to thrombosis. Timely attention can enable prevention and treatment and reduce patient morbidity.

## Data Availability

The datasets presented in this article are not readily available because of ethical restrictions and the protection of patient privacy. Requests to access the data that support the findings of this study should be directed to the corresponding authors.
